# New strategy toward dioxin risk reduction for local residents surrounding severe dioxin hotspots in Vietnam

**DOI:** 10.3402/gha.v6i0.21105

**Published:** 2013-06-20

**Authors:** Tuyet-Hanh Thi Tran, Ngoc-Bich Nguyen, Vu-Anh Le

**Affiliations:** 1 Environmental Health Department, Hanoi School of Public Health, Hanoi, Vietnam; 2 Department of Environmental and Occupational Health, Hanoi School of Public Health and Vietnam Public Health Association, Hanoi, Vietnam

**Keywords:** dioxin hotspots, intervention program, dioxin exposure through foods, risk communication, dioxin risk reduction, Vietnam

## Abstract

**Background:**

A public health intervention program with active involvement of local related stakeholders was piloted in the Bien Hoa dioxin hotspot (2007–2009), and then expanded to the Da Nang dioxin hotspot in Vietnam (2009–2011). It aimed to reduce the risk of dioxin exposure of local residents through foods. This article presents the results of the intervention in Da Nang.

**Methodology:**

To assess the results of this intervention program, pre- and post-intervention knowledge, attitude, and practice (KAP) surveys were implemented in 400 households, randomly selected from four wards surrounding the Da Nang Airbase in 2009 and 2011, respectively.

**Results:**

After the intervention, the knowledge on the existence of dioxin in food, dioxin exposure pathways, potential high-risk foods, and preventive measures significantly increased (*P*<0.05). Ninety-eight percent were willing to follow advice on preventing dioxin exposure. Practices to reduce the risk of dioxin exposure also significantly improved (*P*<0.05). After intervention, 60.4% of households undertook exposure preventive measures, significantly higher than that of the pre-intervention survey (39.6%; χ^2^=40.15, *P*<0.001). High-risk foods had quite low rates of daily consumption (from 0 to 2.5%) and were significantly reduced (*P*<0.05).

**Conclusions:**

This is seen as an effective intervention strategy toward reducing the risk of human exposure to dioxin at dioxin hotspots. While greater efforts are needed for remediating dioxin-polluted areas inside airbases, there is also evidence to suggest that, during the past four decades, pollution has expanded to the surrounding areas. For this reason, this model should be quickly expanded to the remaining dioxin hotspots in Vietnam to further reduce the exposure risks in other areas.

Dioxin is a group of 75 chemicals called polychlorinated dibenzodioxins (PCDDs) that are derived mostly from human sources and can persist in the environment for a long period of time (1). Only 7 of the 75 dioxins have dioxin-like toxicity with 2,3,7,8-tetrachlorodibenzo-*p*-dioxin (2,3,7,8-TCDD), the most toxic man-made chemical (1). Once released into the atmosphere, dioxin is usually bound to particles such as incinerator ash, is shielded from photo-degradation, and stays suspended for a long period of time before settling (2). In water, dioxin accumulates in the bottom sand and sediments of rivers, lakes, and oceans. As dioxin is highly hydrophobic, it is taken up by aquatic organisms and is concentrated as it moves up the food chain to fish and eventually to humans. People can be exposed to dioxin through different pathways, including inhalation, ingestion, and dermal absorption (1).

Currently, there are a large number of studies to examine the health impacts of dioxin on humans, animals, and the ecosystem. Studies on animals show that dioxin exposure affects the hepatic, reproductive, nervous, immune, endocrine, cardiovascular, and pulmonary systems (1). The Institute of Medicine (2011), after reviewing recent scientific publications concerning associations between health outcomes and exposure to 2,3,7,8-TCDD and other chemicals in herbicides used in Vietnam, stated that: ‘sufficient evidence exists to link chronic lymphocytic leukemia (including hairy cell leukemia and other chronic B-cell leukemia), soft-tissue sarcoma (including heart), non-Hodgkin's lymphoma, Hodgkin's disease, and chloracne with dioxin exposure’. There is ‘limited or suggestive evidence of an association’ between exposure and laryngeal cancer; cancer of the lung, bronchus, or trachea; prostate cancer; multiple myeloma; AL amyloidosis; early onset transient peripheral neuropathy; Parkinson's disease; porphyria cutaneatarda; hypertension; ischemic heart disease; type 2 diabetes (mellitus); and spinal bifida in the offspring of exposed people (3).

During the Vietnam War (1962–1971), the US military forces sprayed approximately 76.9 million liters of herbicides as part of the Operation Ranch Hand, including as much as 366 kg of dioxin over Central and South Vietnam. Different tactical herbicides were used, including Agent Orange (AO), Agent White, Agent Purple, Agent Pink, Agent Green and Agent Blue. AO accounted for much of the total chemical sprayed (4) with 1.5 million hectares of land and forests sprayed. Dioxin was a by-product of 2,4,5-T, which was the active ingredient for some of these herbicides (5). Currently, there are 28 identified potential dioxin hotspots in Vietnam. Da Nang Airbase (in the Central of Vietnam) served as a bulk storage and supplies facility for AO and other herbicides during the Vietnam War (the Operation Ranch Hand 1961–1971) and is currently one of the three most severe dioxin hotspots in Vietnam (4–7). Similar to the situation observed in the Bien Hoa dioxin hotspot in the South of Vietnam, local residents living at An Khe, Hoa Khe, Thanh Khe, and Chinh Gian wards, surrounding Da Nang Airbase, are at high risk of exposure to dioxin in soil, water, sand, air, and foods. Potentially high-risk foods are those being grown/raised at areas inside and directly surrounding the Airbase, such as freshwater fish, free-range chickens and ducks, pumpkin, lotus, and so on (6, 8, 9). Both the Bien Hoa and Da Nang dioxin hotspots have received attention from scientists and political leaders in Vietnam and internationally. A number of studies have measured the concentrations of dioxin in the environment, food, blood, and breast milk. Similar to the results taken in Bien Hoa, samples of soil, sediment, blood, and some types of local foods near Da Nang Airbase had elevated levels of dioxin and far exceeded the existing international standards of dioxin in the environment and foods (7, 10, 11). Previous studies have demonstrated that the main source of human exposure is through the consumption of dioxin-contaminated foods, and it was estimated that 95% of background exposure to dioxin is from the food supply (12, 13).

Until 2007, no intervention had been implemented to reduce the risk of exposure for local residents living at these two severe dioxin hotspots. Much attention has been directed toward providing health care and financial supports for AO victims. These include soldiers who served during the War who had diseases associated with dioxin exposure, as well as their children with spinal bifida and other birth defects. Several small-scale pilot remediation projects were also implemented inside the airbases. The local authorities or residents were not aware of the risk of being exposed to dioxin due to the consumption of locally produced high-risk foods. People were not aware of: where the dioxin could be present in the environment; how it could enter into humans; the possible health implications, and how to minimize the risk of exposures (14). Vietnam Public Health Association (VPHA) and its provincial branch in the Bien Hoa dioxin hotspot implemented a pilot public health intervention program during 2007–2009. Following the success of this program in reducing the risk of dioxin exposure for local residents (14, 15), the Ford Foundation continued funding for the expansion of this intervention model to the four wards in the vicinities of the Da Nang Airbase. This intervention program aimed to: (1) improve the knowledge, attitude, and practice (KAP) of local residents in preventing dioxin exposure through foods by implementing information–education–communication (IEC) activities in the community; (2) build capacity for staff and collaborators of Da Nang Public Health Association for the efficient implementation of the intervention program; and (3) strengthen the support and commitment of local leaders in order to develop policies, to assist in the reduction of the risk of exposure to dioxin in contaminated foods for local residents. This article describes the methodology behind the three components of the intervention program, the results of pre- and post-intervention surveys, and provides recommendations for future dioxin risk reduction in other dioxin hotspots.

## Methods

### Public health intervention program

The results of the pre-intervention KAP survey in 2009, as described in ‘Result’ section and experiences from the pilot intervention program implemented in the Bien Hoa dioxin hotspot (2007–2009) (15), were used to develop a public health intervention program. A multi-disciplinary approach and social civil mobilization was developed and implemented in 2010 by VPHA and Da Nang Public Health Association. The intervention program incorporated the following three components: (1) training and increasing KAP for members of Da Nang Public Health Association, representatives of related stakeholders, and collaborators on preventing dioxin exposure through food; (2) implementation of IEC activities to improve KAP on risk reduction of dioxin exposure for local residents living in the four wards, especially for food handlers in households; and (3) policy advocacy to reduce the risk of dioxin exposure through foods for local residents (16).

**Fig. 1:**
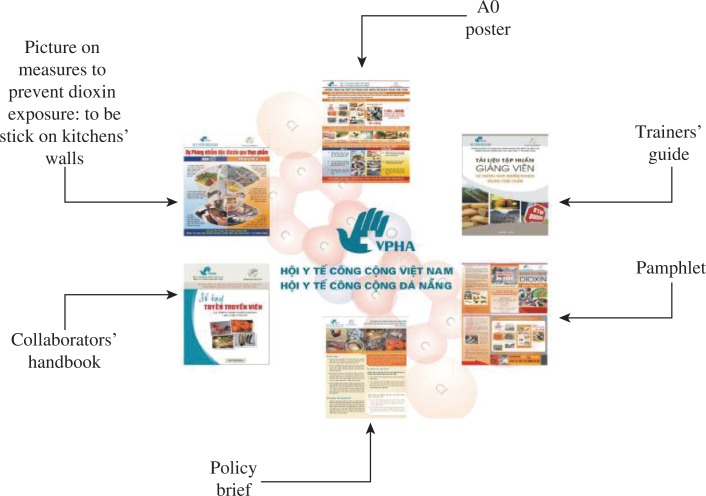
Program's IEC materials in Vietnamese produced by VPHA, 2010 (A0 Poster, Trainers’ Guide; Pamphlet, Policy brief, Handbook for collaborators; Picture).

*Component 1:* Organized training courses for stakeholders, Da Nang Public Health Association's staff and collaborators. Training documents were developed, designed, pre-tested, and produced by VPHA during 2010. The set of training documents included: (1) *Trainers’ Guide – Prevention of Dioxin Exposure through Contaminated Foods*; (2) *The Handbook for Collaborators on Prevention of Dioxin Exposure through Contaminated Foods* (Fig. 1). Two training workshops of trainers (TOT) and collaborators were conducted in 2010. Twenty participants attended the training and five outstanding participants were selected to be trainers in the following training workshops. For the collaborator training, 30 enthusiastic and highly committed collaborators, who were working in the four ward health centers, Women Union, and other mass social organizations were selected and trained in 2010. This training included the nature of dioxin, its health effects, potential high-risk foods, routes of dioxin exposure measures to prevent dioxin exposure through contaminated foods, direct and indirect communication skills at the community, and problem-solving skills when implementing the communication activities at community (16).

*Component 2:* IEC communication in the community. A set of dioxin risk communication documents was developed, designed, tested, and produced. These were based on the results of both the KAP pre-intervention survey and existing documents developed during the intervention program implemented in the Bien Hoa Dioxin hotspot (2007–2009) (14, 17) to ensure that the content and format were suitable for the target audiences. Direct communication activities were conducted in 2010 by the staff of ward health centers, members of the Women's Union and collaborators to provide information and consult food handlers who were responsible for purchasing and preparing food for their families (16). Indirect risk communication activities were frequently conducted on local radio and loudspeakers within six months. Technical staff from VPHA collaborated with the Vietnamese national television channel (VTV2) to produce a documentary film on the intervention program. Journalists and reporters from television stations were also invited to join intervention activities in order to get information. This resulted in media promotion of the intervention program via newspaper articles and news reports (16).

*Component 3:* Policy advocacy. Advocacy activities were conducted in order to call for promulgation of regulations on the prohibition of culturing, harvesting, and consumption of high-risk foods in the intervention areas. A policy brief with recommendations on urgent issues related to dioxin exposure prevention for the local residents in Da Nang in general and for those living in the four program wards in particular was developed, printed, and used (Fig. 1). Advocacy activities also included encouraging local authorities to develop and implement regulations against the actions of catching and selling fish and other aquaculture products taken from dioxin-polluted lakes and ponds near Da Nang Airbase. Besides producing the policy brief, articles and reports on local and national newspapers and radio were also collected to support the policy advocacy campaign (16).

### Assessing the results of the intervention

To assess the results of this intervention program, pre-intervention and post-intervention KAP surveys were implemented using specifically designed questionnaires of approximately 10 pages in length. The surveys were designed to assess changes in the knowledge, attitude, and practices of preventing dioxin exposure through consuming contaminated foods among food handlers in 400 households in four wards located nearby Da Nang Airbase. Sampling frames of the two surveys were the lists of all households living in the four wards in 2009 and 2011 (approximately 16,000 households). Sampling units were households that were systematically randomly selected from the lists. Food handlers, aged 18–65 years from randomly selected households, were invited to participate in the surveys.

Sample sizes for both surveys in the Bien Hoa and Da Nang dioxin hotspots were calculated based on the hypothesized change in health behavior before and after the interventions. As there was no observed reference number from previous studies, the expected estimated change in food safety selection was 50% in total. The sample sizes were estimated, using equation 1:

1 $$n=\frac{p_{0}\left(1-p_{0}\right){\left(Q_{Z}^{\left(1-\alpha \right)}+Q_{Z}^{\left(1-\beta \right)}\sqrt{\frac{p_{\text{A}}\left(1-p_{\text{A}}\right)}{p_{0}\left(1-p_{0}\right)}}\right)}^{2}}{{\left(p_{0}-p_{\text{A}}\right)}^{2}}$$

where *n* is the sample size; *α* is the level of significance; *β* is the power of the study; *p*_0_ is the anticipated population proportion who had safe food selection before the intervention; *p*_A_ was the anticipated population proportion who had safe food selection after the intervention.

We assumed *α*=0.05, *β*=0.9, and that the current and expected population who had safe food selection before and after the intervention was greater than 40%. From equation 1 and using software Power and Sample Size Calculation, it was estimated that 500 or 218 subjects were needed for 50% or 55% improvement in behavior, respectively. A sample size of 400 subjects for each survey was selected to ensure a 50% improvement. This was also the sample size chosen for pre- and post- intervention KAP surveys in the Bien Hoa dioxin hotspot (2007–2009). Data were managed and analyzed using SPSS 17.0 software.

## Results

### Pre-intervention KAP survey

The pre-intervention survey results showed that the knowledge of local residents surrounding the Da Nang dioxin hotspot on the presence of dioxin in the environment was very limited. Of these residents, 76% of respondents were aware that dioxin could be present in untreated water, 54.9% reported that dioxin could be present in soil, 15.9% and 15.3% of respondents knew that dioxin could be present in foods and air, respectively, and 27.6% of people answered that they did not know anything. Respondents’ knowledge of dioxin, potentially high-risk foods and measures to prevent dioxin exposure through reducing the consumption of contaminated foods was also extremely limited. For example, 22.5% of people were aware that freshwater fish and other aquatic products were potentially high-risk foods if cultured in dioxin-contaminated ponds/lakes; 6.1% knew animal fat was potentially a high-risk food if animals were raised in contaminated land; 0.5% knew animal viscera could be a high-risk food and no respondents were aware that eggs and animal milk were high-risk foods if obtained from animals within polluted areas.

While 39.8% of respondents answered that they have already been practicing measures to prevent dioxin exposure through foods, the proportion of those who have been practicing appropriate preventive measures was quite low. For example, 52.8% avoided foods of unclear origin; 15.7% limited their consumption of local freshwater fish and other aquatic products; 11.9% filtered water before use; 3.1% avoided consuming locally cultivated carrot and pumpkin; 1.3% limited the consumption of locally cultured animal fat and viscera and 2.3% of households still consumed their self-cultivated foods. Potentially high-risk foods were consumed frequently on a weekly basis, such as chicken/duck eggs (77%), beef/buffalo meat (65%), fat and lean meat (61.7%), and freshwater fish (51.6%) (17). More details about the comparison of results of pre- and post-intervention surveys are presented in the ‘Results of post-intervention KAP survey conducted in August 2011’ section (17).

The survey results also revealed that conducting direct communication at community meetings, meetings of the Women's Union and other mass social organizations, and the distribution of pamphlets and posters to households, were the preferred IEC communication channels (17). These pre-intervention survey results were shared at a Public Consultation Workshop in Da Nang and this provided an opportunity to mobilize active involvement of related stakeholders in developing a detailed intervention program and implementing activities from the program.

### The risk communication activities

In total, 16,162 households were directly communicated with and consulted in their home by collaborators, and 5,000 households were visited for a second time with an equivalent number of leaflets and pictures distributed. This included 19 reports on local radio and loudspeakers broadcast; 45 community meetings organized to communicate the risk of dioxin exposure and measures to prevent the risk to local residents, and 50 A0-size posters placed at community meeting halls, communal health stations, and at various local markets. The rate of people who have already heard/watched/read about the harmful effects of dioxin on human health was similar to that in the pre-intervention survey (86% in 2009, compared to 86.5% in 2011). There was a statistically significant difference in this proportion among the four wards: 93% of people in An Khe ward, 90.9% in Chinh Gian ward, 84% in Hoa Khe ward, and 75.8% in Thanh Khe Tay ward with χ^2^=17.8, *P*=0.007. The information that people in the four wards were most likely to have heard concerned the health effects of dioxin (46.9%) and measures to prevent dioxin contamination in food (30%). This was important information that people needed to understand during the intervention. About 70% of households in the four intervention wards reported that they have been provided with pictures, to be posted on their kitchen's walls, on measures to prevent dioxin exposure through consumption of contaminated foods. The majority of respondents said that the counseling sessions were informative and easy to understand (83.8%, Table 1). An Khe was the ward with the best communication quality (89.1%), followed by Thanh Khe Tay with 81% of participants rating the content as good/easy to understand. Hoa Khe also had 76.7% and Chinh Gian had 76.2% of respondents who rated the content and quality of communication as good/easy to understand.

**Table 1:** Risk communication activities, Da Nang post-intervention survey, September 2011

Some results of risk communication activities	Da Nang, September 2011
Number of households received direct communication at homes	16,162 households
Number of leaflets and pictures distributed	16,162 leaflets
Number of reportages broadcasted on local radio and loudspeakers	19 reportages
Number of community meetings organized	45 meetings
% Participants already heard/watched/read about dioxin, health effects and preventive measures	86.5
% Participants reported that the counseling sessions by propagandists were informative and easy to understand	83.8

### Results of post-intervention KAP survey conducted in August 2011

**Fig. 2:**
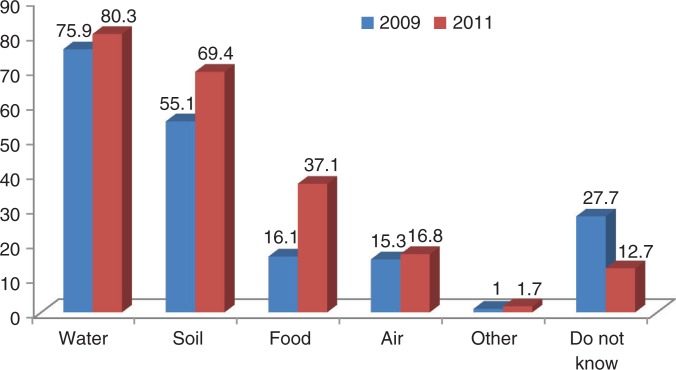
Knowledge of dioxin existence in the environment, Da Nang before and after the intervention, 2009–2011.

*The knowledge* of respondents living in four wards surrounding the Da Nang dioxin hotspot on dioxin and dioxin exposure prevention was significantly improved after the intervention. Generally, more people were aware of where dioxin could be present in the environment (Fig. 2). The proportion of respondents who were aware of dioxin being attached to suspended particles in water bodies in the post-intervention survey was not significantly higher than that of the pre-intervention survey (from 76 to 80.3%, χ^2^=1.79, *P*=0.18); followed by the knowledge of dioxin existence in soil (from 54.9% significantly increased to 69.4%, χ^2^=13.9, *P*<0.01). The knowledge on the existence of dioxin in food increased more than twofold compared to the pre-intervention (16.1–37.1%, χ^2^=35.2, *P*<0.001), while knowledge of dioxin attached to particles in air was increased but was not significantly different to that observed in the pre-intervention (15.3–16.8%, *P*=0.6). The proportion of subjects who did not know where dioxin exists in the environment significantly decreased compared to that in the pre-intervention survey (27.7–12.7%, *P*<0.001).

**Fig. 3:**
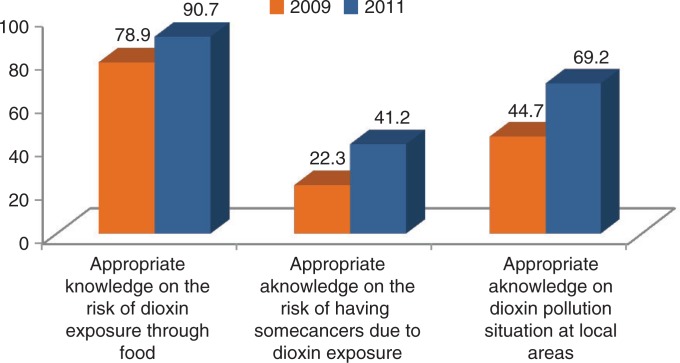
Some appropriate knowledge of local residents on dioxin, Da Nang pre- and post-intervention surveys (2009, 2011).

There was an 11.8% increase to 90.7% in the respondents who believed that dioxin could enter into the human body through food (*P*<0.001, Fig. 3). There was a significant (*P*<0.01, Fig. 3) increase, from 22.3% and 11.8% to 41.2% and 18.2%, respectively in the respondents who knew that cancer and chloracne were associated with dioxin exposure from the pre-intervention survey. There was a significant increase to 69.2% from 44.7% (*P*<0.001, Fig. 3) in the rate of people who thought that the local environment was contaminated with dioxin after the intervention. The rates of people who thought that people can be exposed to dioxin due to consumption of freshwater fish and aquatic products (55.3%), animal fat meat (24%) and eggs, drinking milk as well as egg and milk products (5.9%), were significantly higher than those of the pre-intervention survey, which were 26.1% (*P*<0.001), 10.1% (*P*<0.001), and 0.3% (*P*<0.001), respectively.

Furthermore, the rates of people with improved knowledge concerning high-risk foods as well as measures to reduce dioxin exposure from foods such as fat meat, freshwater fishes, shrimps, crabs, snails, viscera, were significantly increased after the intervention. The rate of people who consume less locally produced freshwater fishes and mollusks from the dioxin-contaminated areas in the post-intervention survey (20.8%) increased by eight times as compared to that before the intervention (2.6%, *P*<0.001). In contrast, the rates of people having knowledge of other risk factors remained relatively low after the intervention. These included: feeding less breast milk a few weeks after birth (0.3%), removing animal fat (5.6%) and eating less egg and dairy products obtained from animals reared in the contaminated areas (16.8%).

There were no significant differences (*P*>0.05) in *Attitude* toward preventing dioxin exposure through consumption of contaminated foods, and the response was generally positive in both pre- and post-intervention surveys. In the post-intervention survey, 86.6% of respondents were confident that they could reduce the risk of dioxin exposure in the contaminated areas, significantly higher than that before the intervention (77.3%; *P*<0.001). The attitude toward the ability to reduce dioxin exposure could be affected by the awareness and educational level of local residents. Only 50% of residents with the lowest educational levels, that is, illiterate or primary education (up to the first grade), believed in the ability to reduce the risk of dioxin exposure. In contrast, residents who were more highly educated, second grade and above had a more positive attitude toward believing in the ability to reduce the risk of dioxin exposure (over 85%, *P*<0.01). Most of the interviewed residents (98%) agreed that they were willing to follow advice to reduce their risks of dioxin exposure (Table 2). This was not significantly higher than that of the pre-intervention survey (97%). Before the intervention, 96.9% of respondents were willing to quit some of their favorite foods if they knew that these foods were at high-risk of being contaminated with dioxin and was equivalent to the pre-intervention result (95.8%). There was a statistically significant difference between males (95.1%) and females (98.4%) who were willing to give up their favorite foods (χ^2^=8.23; *P*<0.05). The number of respondents (95.1%) who were willing to buy uncontaminated foods with higher prices was not significantly higher compared to those in the pre-intervention survey (92.1%) (χ^2^=5.3; *P*=0.07). The average additional amount of money that people were willing to pay to buy fresh food was in the range 5,000–10,000 VND/household/day (approximately 0.25–0.5 USD/household/day).

**Table 2:** Positive attitude toward dioxin exposure risk prevention, Da Nang pre- and post-intervention

Attitude	Da Nang pre-intervention 2009 (%)	Da Nang post-intervention 2011 (%)	χ^2^, *P*
Confident in the possibility to reduce the risk of dioxin exposure in the contaminated areas	77.3	86.6	χ^2^ *=*15.3; *P*<0.001
Willing to follow advices to reduce their risks of dioxin exposure	97	98	χ^2^=4.5; *P*=0.2
Willing to quit some favourite foods if aware the foods were at high risk of being contaminated with dioxin	96.9	95.8	χ^2^=4.6; *P*=0.2
Willing to buy uncontaminated foods with higher prices	92.1	95.1	χ^2^=5.3; *P*=0.07

**Fig. 4:**
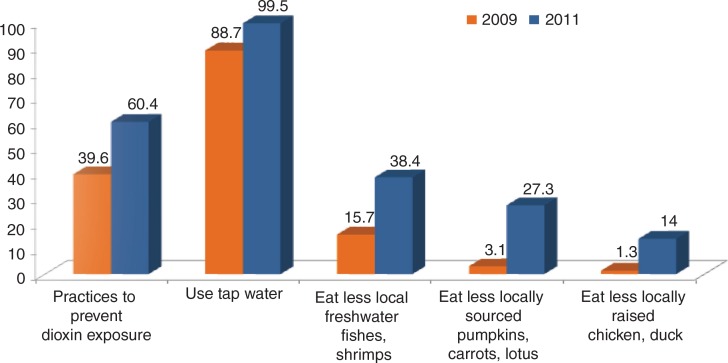
Some practices to prevent dioxin exposure undertook by local residents living in Da Nang Dioxin hotspot, pre- and post-intervention surveys (2009, 2011).

*Practices* to prevent dioxin exposure through consumption of contaminated foods were also improved after the intervention and generally better than the results of the post-intervention survey in Bien Hoa (15). Specifically, 99.5% of households used tap water for drinking and domestic consumption purposes, significantly increased compared to that in the pre-intervention (88.7%, *P*<0.001, Fig. 4). Four households (1%) were still consuming locally self-cultivated foods: two households in the Hoa Khe Ward and two households in the Thanh Khe Tay Ward. Following the intervention, 99% of the households did not use, consume, or sell locally self-cultivated foods and 67.2% of respondents did pay attention to food sources. The factors to which local people paid most attention to when they purchased foods were ‘cleanness, freshness, safety’ (88.9%); ‘deliciousness’ (4.9%); ‘cheap’ (2%); ‘nutritional value’ (0.5%), and ‘other factors’ (3.5%). There was a significant correlation between the practice of paying attention to food sources when buying and both the awareness of dioxin contamination in living areas and the awareness of dioxin exposure risks via consumption of contaminated food. The rate of having appropriate awareness of dioxin contamination in the locality and dioxin exposure routes through contaminated foods in the group already undertaking exposure preventive measures (79.4% and 72%) were significantly higher than that observed in the group without any exposure preventive measures (38.1% and 64.7%). There was a significant increase to 60.4% of households that already undertook exposure preventive measures, compared to that of the pre-intervention survey (39.6%) (Fig. 4).

**Fig. 5:**
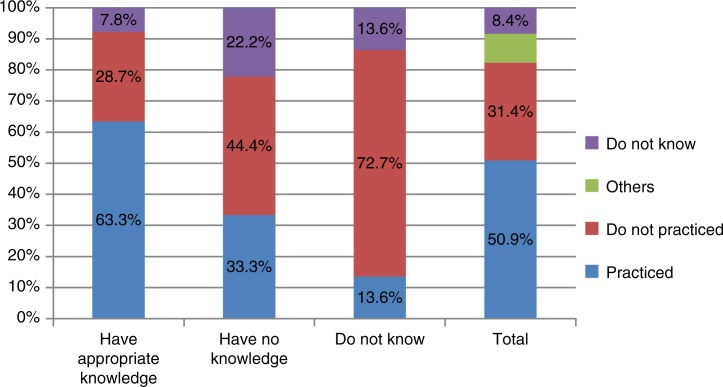
The relationship between knowledge on the risks of dioxin exposure through consuming contaminated foods and practices of preventing dioxin exposure, Da Nang 2011.

The results also showed that locally produced foods at high dioxin contamination risks such as freshwater fishes, chicken eggs, duck eggs, pumpkin had quite low rates of daily consumption (≤2.5%) and were significantly lower than those of the pre-intervention survey. Foods with high consumption frequencies on a weekly basis included mustard greens (79.4%), katuk (77.2%), cattle meat (70.1%), water spinach (60.8%), and lean pork (62%) The weekly consumption frequencies of some high-risk foods decreased significantly compared to those in the pre-intervention survey. From 2009 to 2011, the following reduction in consumption was observed: pumpkin on a weekly basis from 63.7 to 34.5%; freshwater fish from 51.4 to 17.4%; wild goose, duck meat from 15.3 to 5.6%, and viscera from 6.3 to 2.9%. The knowledge of respondents on the risk of exposure via foods was significantly related to practices of exposure prevention. The proportion of people with the correct knowledge who implemented measures to prevent dioxin exposure (63.3%) was higher than in the group with inappropriate knowledge (33.3%) and the group who responded ‘have no idea’ (13.6%) (Fig. 5).

## Discussion

Although village meetings, loudspeakers, health personnel, direct communication through household counseling visits, pamphlets and pictures were not the preferred communication methods, they were deliberately selected in the program due to the political and socially sensitive nature of the dioxin contamination issue in Vietnam. These direct communication methods targeted only at-risk localities, that is the local residents living in areas surrounding the Da Nang Airbase, without terrifying people living in Da Nang City or causing adverse impacts on the local social, economic, and political situations. The post-intervention survey showed that approximately 70% of households in the four intervened wards in Da Nang have been provided with pictures on measures to prevent dioxin exposure through consuming contaminated foods and was higher than Bien Hoa after the intervention (66%). Among households who have been provided with pictures, 94.3% had already posted the picture on their kitchen wall. This was considered an important output of the program and conveniently helped people to remember and refer to the information. By July 2011, 72.8% of households still retained the pictures on measures to prevent dioxin exposure through limiting consumption of contaminated food at homes.

The results showed that, similar to the situation assessed in Bien Hoa (14), before implementing the intervention program, the knowledge of local residents surrounding Da Nang on dioxin and measures to prevent dioxin exposure through consuming contaminated foods was extremely limited. This was particularly true of their knowledge on the presence of dioxin in locally cultivated foods and dioxin exposure pathways. The knowledge of people in the four wards around Da Nang airport in the post-intervention survey was significantly improved and higher than those of the pre- and post-intervention surveys in Bien Hoa (2007–2009). Specifically, the proportion of people with accurate knowledge of the presence of dioxin in water, soil, food, and air in the four wards near Da Nang airport post-intervention was 80.3, 69.4, 37.1 and 16.8%, which were higher compared with the pre-intervention results of 76, 54.9, 16.1 and 15.3%, respectively. These were also higher than the results of the post-intervention survey in Bien Hoa in 2009 of 61.7, 49.5, 34, and 14.7%, respectively. The rate of respondents who answered ‘do not know’ in the post-intervention survey in Da Nang (12.7%) was lower than the results in Bien Hoa (16.9%). According to the International Agency for Research on Cancer (1997), once released into the environment, dioxin can present itself in soil, water, air, and foods. Studies recently conducted in Vietnam also found that almost 40 years after the war, the concentrations of dioxin in the environment, especially in soils, sediments, and in some types of local foods such as ducks, freshwater fish, toad, free-range chicken, and so on in hotspots were still very high (7, 9, 10, 18–20).

Comparison of the pre- and post-intervention surveys’ results showed that most of respondents (98%) in the four intervention wards in Da Nang reported that they were ready to follow the recommendations to reduce the risk of dioxin exposure. This was higher than the results of the post-intervention survey at Trung Dung and Tan Phong wards in Bien Hoa in 2009 (96%) (15). The results of the post-intervention survey also showed that the knowledge and practices of local residents at the four intervention wards on preventing the risk of dioxin exposure were significantly correlated. Thus, the intervention measures for improving the knowledge of dioxin existence in the environment among the people in the four wards of Da Nang City as compared with the pre-intervention were important in promoting preventive behaviors. The positive changes in the behavior of the local residents surrounding Da Nang played a significant role in reducing their risks of dioxin exposure in the environment, especially from foods. This survey was undertaken less than one year post-intervention, while the process derived from receiving improved knowledge to change in attitude to practice appropriate behavior usually takes a longer time (21). Therefore, it is expected that the practices of local residents in reducing their risks of being exposed to dioxin in foods will be improved even further in the coming years. This assumption will be verified in a study to be undertaken during 2013–2014 to assess the sustainability of the program after three and five years post-cessation in Da Nang and Bien Hoa dioxin hotspots.

Regarding the change in weekly food consumption frequency, nutritionists may express their concerns regarding the significant reduction in the proportion of households having freshwater fish at least once from 51.4% in 2009 to 17.4% in 2011 (χ^2^=103.5, *P*<0.001) as it is recognized that inclusion of fish in the diet is important. However, it should be noted that the survey showed that the proportion of people having seafood weekly increased significantly from 38.3% in 2009 to 51.2% in 2011 (χ^2^=13.5, *P*<0.001). As Da Nang is located by the sea, the increased awareness of the risk of exposure to dioxin in local freshwater fish may have prompted a change to seafood instead, resulting in positive impacts on diet.

On 9 August 2012, the US Agency for International Development and the Vietnamese Ministry of National Defense jointly launched an estimated USD 43 million project to clean up the polluted soil in Da Nang Airbase, to help solve the problem inside the airbase and prevent further expansion of the toxic chemical to the surrounding areas. However, in the four decades since the War ended, dioxin pollution has extended outside the Bien Hoa and Da Nang airbases (7, 22, 23). Thus, the Public Health intervention program was perceived as a complementary and effective intervention model to help in the reduction of exposure risk to dioxin in the environment, especially in foods. As a consequence, it should be expanded to the remaining dioxin hotspots in Vietnam to prevent further exposure of local people to dioxin.

## Conclusion and recommendations

Due to the sensitivity of dioxin contamination and the fact that only certain localities remain polluted, direct communication channels toward people at risk, although not the preferred communication channels, should still be applied. The public health intervention program implemented in Da Nang dioxin hotspot has shown initial success in improving knowledge, attitudes and practices among local people in the introduction of measures to reduce the risk of dioxin exposure through the consumption of contaminated foods. Proportionally high numbers of local residents were aware of health risks associated with dioxin exposure and understood and applied recommended methods to prevent dioxin exposure through food. The consumption frequencies of some high-risk foods decreased significantly compared with those of the pre-intervention survey. Most of the surveyed households commented that the counseling information provided by collaborators in this program was easy to understand and contained good content. In addition to direct and indirect communication activities, the regulations against culturing, harvesting, consuming and selling high-risk foods in the dioxin-polluted areas in Da Nang City must be strictly implemented as soon as possible because the actions of catching fish, prawns, and crabs were still observed in some dioxin-contaminated lakes surrounding Da Nang Airbase. This will prevent fishermen from other districts in the City coming to these lakes to fish. While greater effort is required for dioxin remediation activities at the remaining 28 identified potential dioxin hotspots in Vietnam, this Public Health intervention program should be expanded to other dioxin hotspots to further limit exposure of people to dioxin. This is of particular importance as in the past four decades, dioxin pollution has expanded to areas surrounding the airbases while remediation efforts only focus on areas inside the airbases. An assessment on the sustainability of this intervention program will be undertaken in 2013 to provide evidence for sustainable expansion of the model to other dioxin hotspots in the country.

## References

[1] Center for Health Environment and Justice (1999). The American people's dioxin report – technical support document.

[2] International Agency for Research on Cancer (1997). IARC monographs on the evaluation of carcinogenic risks to humans.

[3] Institute of Medicine Committee to Review the Health Effects in Vietnam Veterans of Exposure to Herbicides (2011). Veterans and Agent Orange: update 2010.

[4] Stellman J, Stellman S, Christian R, Weber T, Tomasallo C (2003). The extent and patterns of usage of Agent Orange and other herbicides in Vietnam. Nature.

[5] Young AL (2009). The history, use, disposition and environmental fate of Agent Orange.

[6] Wayne Dwernychuk L (2005). Dioxin hot spots in Vietnam. Chemosphere.

[7] Hatfield Consultants (2006). Assessment of dioxin contamination in the environment and human population in the vicinity of Da Nang airbase, Vietnam – Report 2: draft final sampling Design.

[8] Tuyet Hanh TT, Vu Anh L, Ngoc Bich N, Tenkate T (2010). Environmental health risk assessment of dioxin exposure through foods in a dioxin hot spot – Bien Hoa City, Vietnam. Int J Environ Res Public Health.

[9] Schecter A, Quynh H, Pavuk M, Papke O, Malish R, Constable J (2003). Food as a source of dioxin exposure in the residents of Bien Hoa City, Vietnam. J Occup Environ Med.

[10] Hatfield Consultants and Office of the National Steering Committee 33 MONRE (2009). Comprehensive assessment of dioxin contamination in Da Nang airport, Vietnam: environmental levels, human exposure and options for mitigating impacts.

[11] Schecter A, Hoang TQ (1995). Agent Orange and Vietnamese: the persistence of elevated dioxin levels in human tissues. Am J Public Health.

[12] Institute of Medical Research (2004). Dioxin and dioxin-like compounds in the food supply: strategies to decrease exposure.

[13] Dwain W (2002). Dioxin intake from food: current state of knowledge and uncertainties in assumptions.

[14] Vu Anh L, Ngoc Bich N, Thanh Ha N, Duc Minh N, Minh Son D, Tuyet Hanh Tran T (2008). Knowledge, attitude and practice of local residents at Bien Hoa City – Vietnam on preventing dioxin exposure through foods. Organohalogen Comp.

[15] Vu Anh L, Tuyet Hanh T, Ngoc Bich N, Duc Minh N, Thanh Ha N (2010). Public health intervention program to reduce the risk of dioxin exposure through goods in Bien Hoa City Vietnam – encouraging results after one year of intervention Organohalogen Comp.

[16] Vu-Anh L, Tuyet-Hanh T, Ngoc-Bich N, Duc-Minh N, Thanh-Ha N, Kim-Ngan N (2011). Public health intervention program to reduce the risk of dioxin exposure through foods in Da Nang hot spot. Organohalogen Comp.

[17] Vu Anh L, Tuyet Hanh Tran T, Ngoc Bich N, Duc Minh N, Thanh Ha N, Kim Ngan N (2010). Knowledge, attitude and practices of local residents at four wards, Da Nang City – Vietnam on preventing dioxin exposure through foods. Organohalogen Comp.

[18] Schecter A, Pavuk M, Constable JD, Dai LC, Päpke O (2002). A follow-up: high level of dioxin contamination in Vietnamese from Agent Orange, three decades after the end of spraying. J Occup Environ Med.

[19] Hatfield Consultants and Office of the National Steering Committee 33 MONRE (2011). Environmental and human health assessment of dioxin contamination at Bien Hoa airbase, Vietnam.

[20] Hatfield Consultants Ltd and Viet Nam – Russia Tropical Centre (2009). Evaluation of contamination at the Agent Orange dioxin hotspots in Bien Hoa, Phu Cat and Vicinity, Vietnam.

[21] Oldenburg B, Ffrench M, Glanz K (1999). The application of staging models to the understanding of health behaviour change and the promotion of health. Psychol & Health.

[22] Minh NH, Minh TB, Kajiwara N, Kunisue T, Iwata H, Viet PH (2007). Pollution sources and occurrences of selected persistent organic pollutants (POPs) in sediments of the Mekong River delta, South Vietnam. Chemosphere.

[23] Minh NH, Son LK, Nguyen PH (2008). Dioxin contamination in Bien Hoa Airbase and its vicinities: environmental levels and implication of sources. Organohalogen Comp.

